# Detection of Pan drug resistance OXA-48 producing Providencia in an ICU patient for the first time in Nepal

**DOI:** 10.1186/s13756-019-0608-1

**Published:** 2019-10-15

**Authors:** Ranjit Sah, Shusila Khadka, Gentle Sunder Shrestha, Subhash Acharya, Diptesh Aryal, Pramesh Shrestha, Hari Prasad Kattel, Niranjan Prasad Shah, Bharat Mani Pokhrel, Yogendra Prasad Singh, Basista Rijal, Hakan Erdem

**Affiliations:** 1 Department of Microbiology, Tribhuvan University Teaching Hospital, Institute of Medicine, Kathmandu, Nepal; 2 Department of Anesthesiology (ICU), Tribhuvan University Teaching Hospital, Institute of Medicine, Kathmandu, Nepal; 3 Research Department, Institute of Medicine, Kathmandu, Nepal; 4 IDI-IRI, Ankara, Turkey

**Keywords:** OXA-48, Drug resistance, *Providencia* species, Nepal

## Abstract

**Background:**

Resistance to antimicrobial agents of pathogenic bacteria has become a major problem in routine medical practices. Carbapenem resistance has long been increasing. The production of carbapenem- hydrolysing β-lactamases (carbapenamases), which include NDM, KPC, OXA-48, IMP-1 and VIM is the most common mechanism.

**Case presentation:**

A 56 years old male presented with fever and mental changes with progressively decreasing sensorium for the last 3 days. He was admitted to Intensive care unit (ICU) with a diagnosis of meningoencephalitis. On day seven, he developed ventilator associated pneumonia due *Klebsiella pnemoniae* and *Acinetobacter baumannii*. He was on meropenem, but the isolates were susceptible to colistin, tigecyclin and amikacin solely. Hence, amikacin was started with addition of intravenous and nebulized colistin. Subsequently, vital signs improved with resolution of fever. However, on day 18, he developed fever once again with a drop in blood pressure. Inotropic support was maintained, and echinocandins and tigecycline were added to the regimen.

Repeat blood and urine culture grew *Providencia* species, which were resistant to most of the drugs on phenotypic Kirby-Bauer disk diffusion method and are intrinsically resistant to colistin and tigecycline. Phenotypic detection of ESBL (combined disk method), MBL, KPCs, AmpC and co-producer were tested according to updated CLSI guideline and all were negative. But the Modified Hodges test was found to be positive. Consequenty, OXA-48 drug resistance pattern was brought into action by blank disc method according to A Tsakris et al., which revealed indentation of growth toward both EDTA and EDTA/PBA disk indicating production of OXA-48 carbapenamase. To confirm the resistance pattern we processed the isolated colonies for Xpert Carba-R (Cepheid) assay, which detected blaOXA-48 gene and confirmed the OXA-48 drug resistance pattern. Hence, the infecting organism was not susceptible to any of the antibiotics. The patient was kept under isolation and on 31th day of admission, he died of septic shock.

**Conclusions:**

Carbapenamase production along with intrinsic colistin resistance in infecting bacterial pathogens can cause fatal outcomes in the resource limited countries like Nepal where new antibiotic combinations ceftazidime+ Avibactam, or aztreonam +avibactam are not available. Drug resistance patterns including OXA 48 producer should be characterized in all cases by standard phenotypic methods or by Xpert Carba-R assay and larger studies are required to know the exact burden of OXA 48 producer in Nepal.

## Background

The development of antibiotics remains one of the most significant advances in modern medicine. Antibiotics have saved countless lives and continue to be the mainstay of therapy for bacterial infections. The clinical success of the first β-lactam, penicillin G (benzylpenicillin), prompted the search for and development of additional derivatives. This quest gave rise to the β-lactam antibiotics in clinical use today (penicillins, narrow- and extended-spectrum cephalosporins, monobactams, and carbapenems) [1, 2]. Unfortunately, β-lactamase-mediated resistance to β-lactam antibiotics emerged as a significant clinical threat to these lifesaving drugs.

There are two globally accepted classification schemes for β-lactamases, the first one is based on amino-acid sequence classification and the second one is based on functionality. β-lactamases were divided into four classes (Class A–D) based on their sequence similarity by Ambler in 1980. Classes A, C and D function by the serine ester hydrolysis mechanism, whereas class B β-lactamases, also known as metallo β-lactamases, have a zinc ion participating in catalysis [3–5]. The classification scheme by functionality was purposed by Bush et al. in 1995 and was updated in 2009 by Bush-Jacoby group. It takes into account the substrate and inhibitor profiles in an attempt to group the enzymes in ways that can be correlated with their phenotypes in clinical isolates [6]. The updated system includes group 1 (class C) cephalosporinases; group 2 (classes A and D) broad-spectrum, inhibitor-resistant, and extended-spectrum β-lactamases and serine carbapenamases; and group 3 metallo-β-lactamases, each of which is also divided into several different subgroups [6]. Awareness is required to all infectious diseases (ID) and general physicians since penicillins, cephalosporins, and carbapenems are included in the preferred treatment regimens for many infectious diseases due to the fact that presence and characteristics of these enzymes play a critical role in the selection of appropriate therapy [6].

Resistance to antimicrobial agents of pathogenic bacteria has become a major problem in current medical practices. Over the last decades, carbapenems have been used as the last-resort drugs in the treatment of serious nosocomial infections caused by multidrug-resistant Gram-negative bacteria. However, carbapenem resistance has been increasing and the most common mechanism is the production of carbapenem-inactivating β-lactamases (carbapenamases) [7]. Here, we report an OXA-48 drug resistance pattern observed in blood and urinary isolates of *Providencia* species in a fatal meningoencephalitis case who subsequently developed ventilator associated pneumonia and urinary tract infection.

## Case presentation

A 56 years old male from Parsa (district) in Nepal presented to emergency department with fever and altered conscious for the last 3 days. His sensorium was progressively worsening, and thus, he was admitted to intensive care unit (ICU) with a diagnosis of meningoencephalitis. The patient was intubated at ICU admission, on day seven he developed ventilator associated pneumonia. *Klebsiella pneumoniae* and *Acinetobacter baumannii* were isolated from the sputum sample. He was on meropenem, but the isolates were susceptible to colistin, tigecycline and amikacin solely (beta-lactam antibiotics, fluroquinolones, doxycycline, gentamicin and cotrimoxazole were resistant on phenotypic Kirby-Bauer disk diffusion method). Hence, amikacin was started with addition of intravenous and nebulized colistin. Subsequently, vital signs improved with resolution of fever. However, on day 18, he was febrile once again with drop in blood pressure. Inotropic support was maintained and both echinocandins and tigecycline were added to the regimen.

Although blood and urine cultures grew *Providencia* species, sputum samples were sterile. Microscopic examination of urine revealed plenty of pus cells, few epithelial cells and no erythrocytes. Patient was at septic shock and he was diagnosed as meningoencephalitis with overlapping ventilator associated pneumonia and urinary tract infection.

**Fig. 1:**
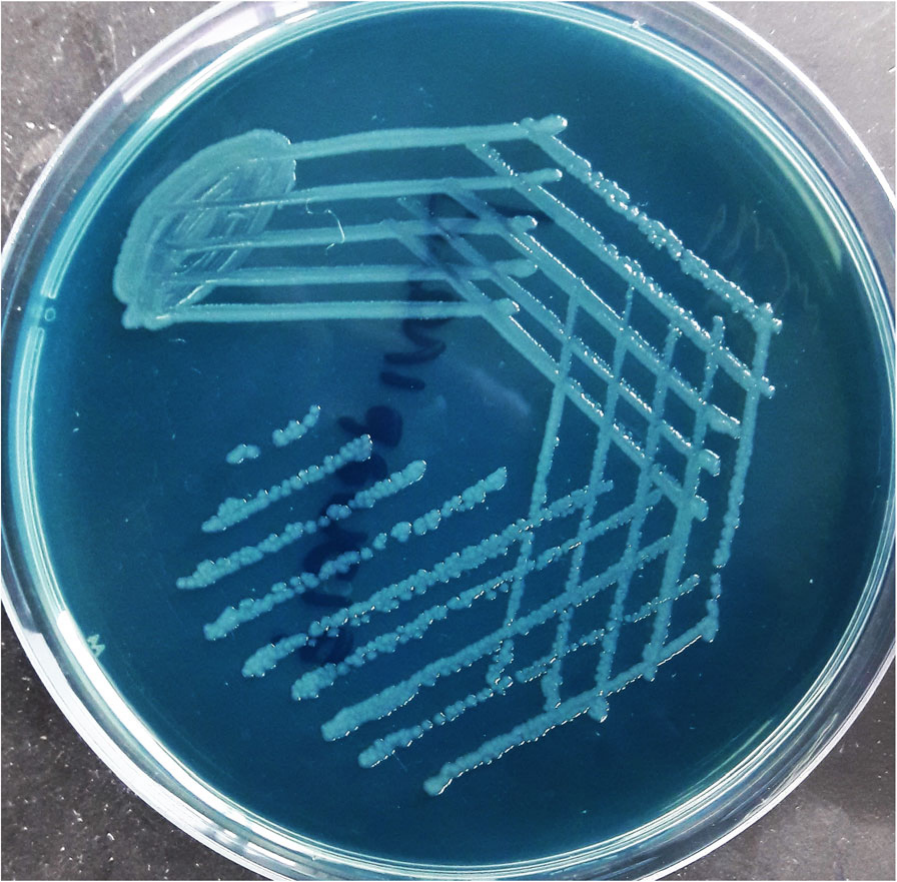
non-lactose fermenting colonies of Providencia vermicola on CLED (Cystine Lactose Electrolyte Deficient) agar

**Fig. 2:**
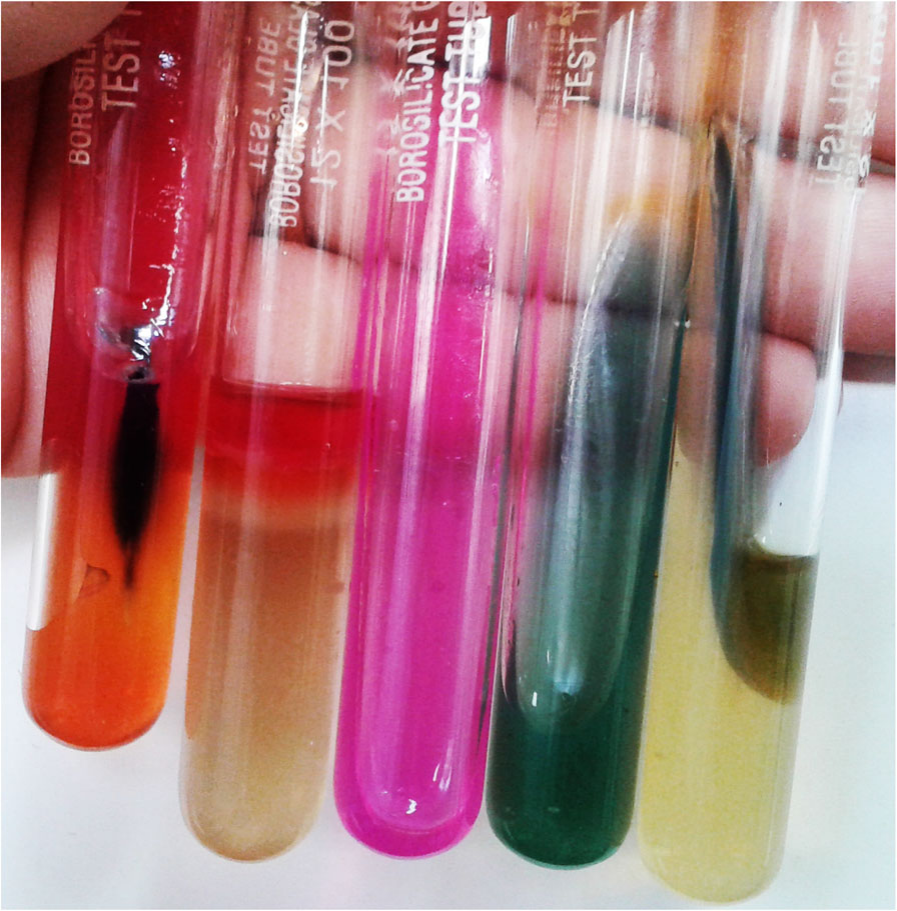
Biochemical tests showing the organism belonging to Providencia species

The recovered *Providencia* isolates were resistant to most of the drugs including colistin (intrinsic resistance). Culture of blood and urine revealed gram negative bacilli with non-lactose fermenting colonies on MacConkey and CLED (Cystine Lactose Electrolyte Deficient) agar (Fig. 1) respectively. The organism fermented glucose, but could not utilize lactose and sucrose on sugar fermentation test. Sulphite indole motility test revealed motile indole positive organism. Citrate utilization and urea hydrolysis tests were positive. To confirm that the organism belongs to the tribe Proteeae of family Enterobacteriaceae, phenyl pyruvic acid test was performed which was found to be positive (Fig. 2). On the basis of aforementioned phenotypic tests, *Providencia* species were identified. Antibiotic susceptibility testing was done by Kirby Bauer method according to CLSI (Clinical & Laboratory Standards Institute) [8] which revealed pan-drug resistant isolate (resistance to almost all drugs available in the laboratory like amoxycillin/ampicillin, ciprofloxacin, levofloxacin, amikacin, gentamicin, meropenem, imipenem, erythromycin, clindamycin, ceftriaxone, ceftazidime, cefoperazone-sulbactam, ampicillin-sulbactum, chloramphenicol, tetracycline, doxycyclin and since *Providencia* species belongs to tribe Proteeae of family Enterobacteriaceae, they are intrinsically resistant to colistin and tigecycline and therefore drug susceptibility testing is not recommended for this group of organisms).

**Fig. 3:**
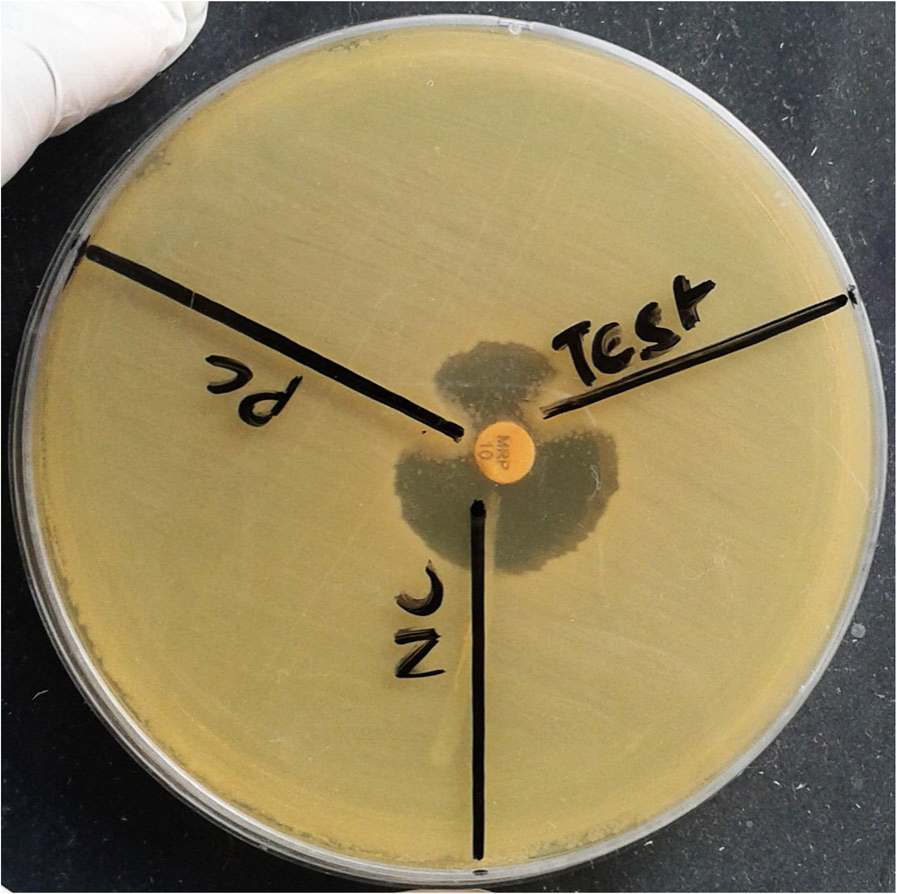
Modified Hodge Test positive for test organism (Providencia)

**Fig. 4:**
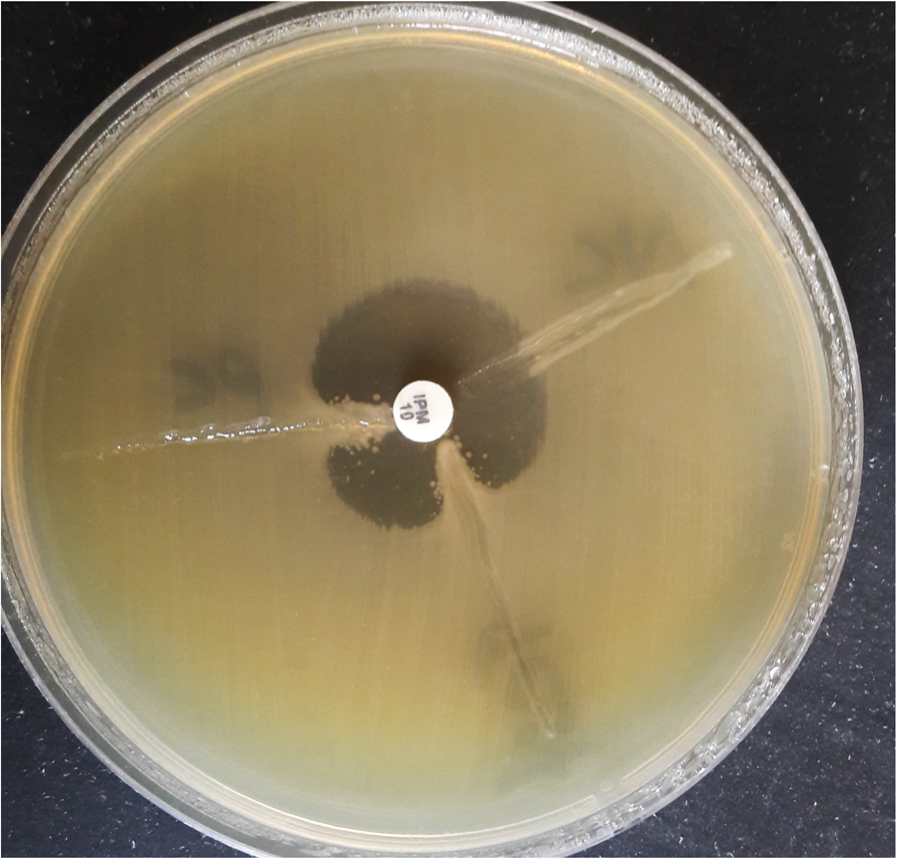
Modified Hodge Test positive for test organism (Providencia)

**Fig. 5:**
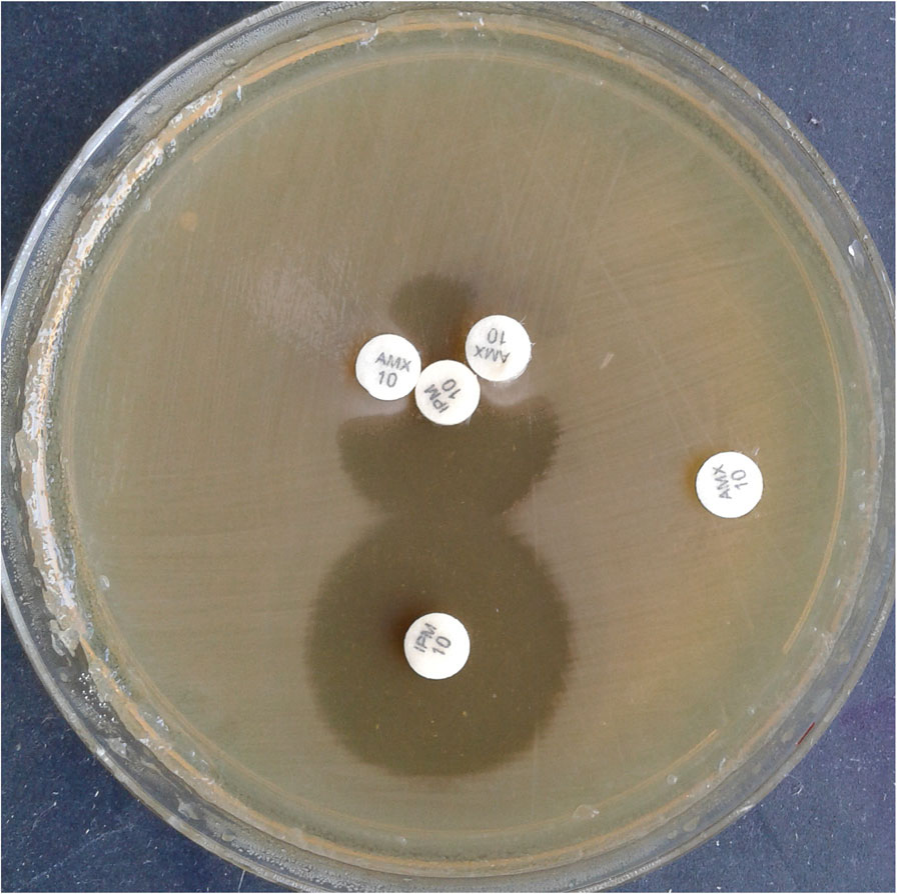
Detection of OXA-48 by using antibiotic containing disk which is intrinsically resistant to Providencia instead of Blank Disk3. {E – EDTA-0.1 M 10 μl (292 μg of EDTA), P-Phenyl Boronic Acid- 10 μL (containing 600 μg of PBA)}3

**Fig. 6:**
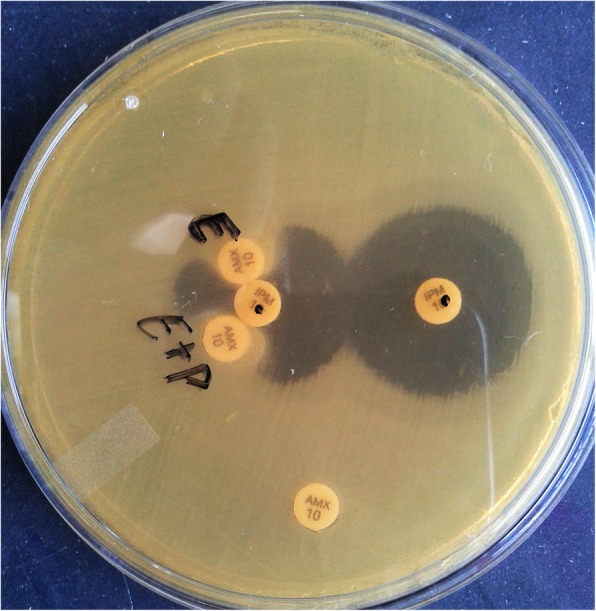
Detection of OXA-48 by using antibiotic containing disk which is intrinsically resistant to Providencia instead of Blank Disk3. {E – EDTA-0.1 M 10 μl (292 μg of EDTA), P-Phenyl Boronic Acid- 10 μL (containing 600 μg of PBA)}3

**Fig. 7:**
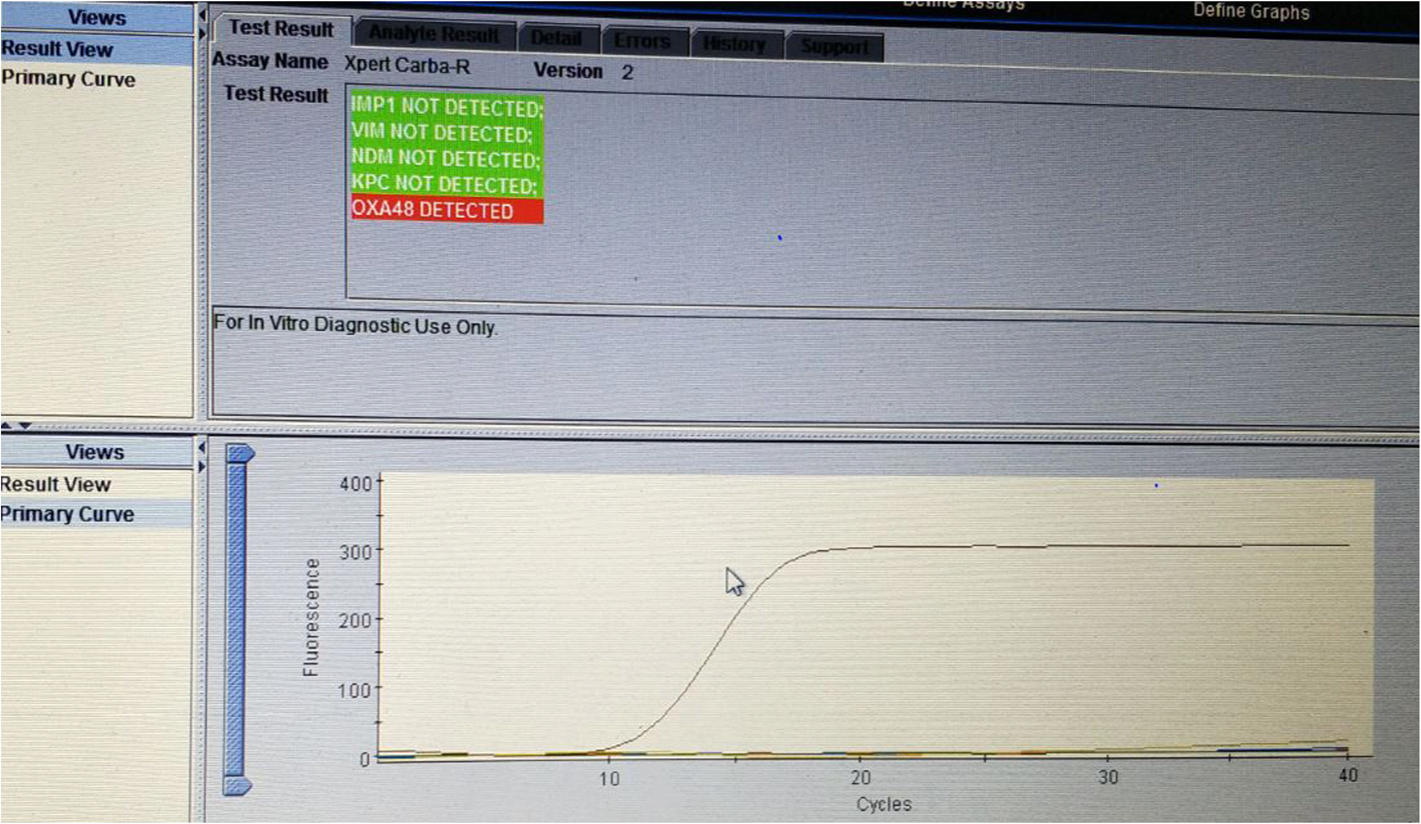
Cepheid Xpert Carba-R Assay showing detection of blaOXA-48 gene sequences

**Fig. 8:**
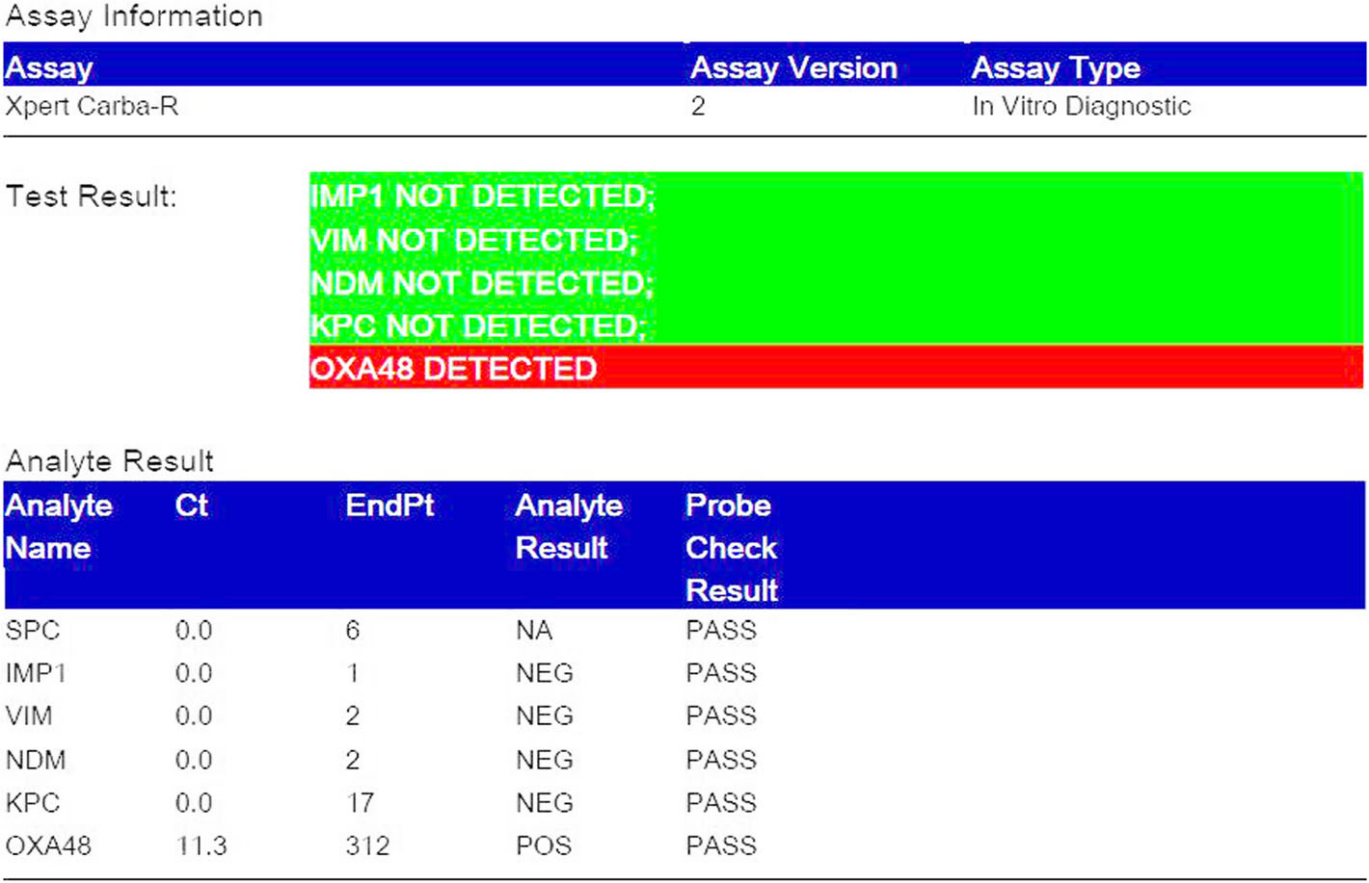
Cepheid Xpert Carba-R Assay showing detection of blaOXA-48 gene sequences

To characterize the drug resistance pattern, phenotypic detection of ESBL and Modified Hodge test were done according to CLSI guideline [8]. ESBLs were tested by combination disk method in which ceftazidime (CAZ) and ceftriaxone (CTX) alone and in combination with clavulanic acid (CA) (10 μg) were used. An increase in zone of inhibition of more than or equal to 5 mm for either antimicrobial agent in combination with CA versus its zone when tested alone were negative, but Modified Hodges test revealed carbapenamase producing bacteria (Figs. 3 and 4). Subsequently, phenotypic detection of MBL, KPCs, AmpC and co-producer were tested according to CLSI guideline and all were negative. On the background of positive Modified Hodge test and negative ESBL, MBL, KPCs, AmpC and co-producer, OXA-48 drug resistance pattern was brought into action. We used blank disc method for this purpose according to A Tsakris et al. [9] which revealed indentation of growth toward both EDTA and EDTA/PBA disc (Figs.5 and 6) indicating production of OXA-48 carbapenamase. The isolates were transported to India, where they were confirmed as *Providencia vermicola* by VITEK MS, which use matrix-assisted laser desorption/ionization time-of- flight (MALDI-TOF) technology. Added to that, the resistance pattern of the isolated colonies was processed by Xpert Carba-R (Cepheid) assay, which detect blaOXA-48 gene (Figs. 7 and 8) and OXA48 drug resistance pattern was confirmed. The patient was kept under isolation. On 31th day of admission he died of septic shock and lack of other antibiotic option (intrinsic resistance to colistin and unavailability of ceftazidime+ avibactam or aztreonam +avibactam combination in both Nepal and India for testing in laboratory or for systemic use in patient).

## Discussion and conclusion

Among the carbapenamase-producing Enterobacteriaceae species, production of OXA-48 can be suspected when the Modified Hodge Test (MHT) is positive and the inhibitor-based combined disk tests for KPC and MBL production are negative. This phenotypic test (OXA-48 disk test) provided a straightforward, convenient, and accurate means of detection of OXA-48 carbapenamase in organisms exhibiting reduced susceptibility to carbapenems [9]. In developing countries where genetic confirmation of the isolates and detection of drug resistance gene is not available, phenotypic detection by standard methods and genetic confirmation by Xpert Carba-R assay is a proper alternative. Cepheid Xpert Carba-R assay is a qualitative, on-demand real-time PCR test for rapid detection and differentiation carbapenamase producing organism by detecting the blaKPC, blaNDM, blaVIM, blaIMP-1 and blaOXA-48 gene sequences from pure colonies encoding the KPC, NDM, VIM, IMP-1, and OXA-48 enzymes respectively and helps clinicians to optimize and direct therapeutic strategy (antibiotic therapy) for patient management [10]. In our case we have first performed phenotypic testing as described by Tsakris et al. [9] and subsequently confirmed OXA-48 enzyme by detecting the blaOXA-48 gene by Xpert Carba-R assay.

The first identification of OXA-48-producing Enterobacteriaceae was in an isolate in Turkey in 2001 [11, 12]. Shortly thereafter, there was an outbreak of OXA-48-producing *Klebsiella pneumoniae* isolates reported in Istanbul in 2006 [12, 13]. Since its recognition, there have been increasing numbers of reports of OXA-48-producing organisms worldwide. Avibactam in combination with either of ceftazidime, cefepime, aztreonam, imipenem, or meropenem can inhibit OXA-48-producing Enterobacteriaceae and are drug of choice [13]. Among them ceftazidime/avibactam has proven to be most successful combination [13]. International Network for Optimal Resistance Monitoring (INFORM) global surveillance study in 2012 to 2015 suggested that the in vitro activity of ceftazidime-avibactam was better against the subset of metallo-β-lactamase (MBL)-negative, OXA-48- and OXA-48-like-positive isolates (99.2 and 100% susceptible, respectively) [14]. Vasoo et al. also emphasized that ceftazidime-avibactam was active against all KPC-, IMI-, SME-, and most OXA-48 group-producing isolates (93%), but not to metallo-β-lactamase producers [15].

Sader et al. they analysed in vitro activity of aztreonam-avibactam against a large collection (2016) clinical Enterobacteriaceae isolates recovered from patients hospitalized in U.S. medical centers, as well as selected carbapenemase (NDM-like and OXA-48-like)-producing Enterobacteriaceae isolates recovered outside the United States. They found aztreonam-avibactam was very active against KPC, MBL and OXA-48-like producers. Among 57 OXA-48-producing isolates obtained from outside the US, two were sensitive with MIC ≤0.03 μg/ml, two were sensitive with a MIC of 0.06 μg/ml, and 17 were sensitive with MIC of 0.12 μg/ml, 32 were sensitive with MIC 0.25 μg/ml and four were sensitive with MIC of 0.5 μg/ml. All OXA-48-like producers (100%) were sensitive at MIC 0.5 μg/ml. They also tested against the 63 strain of MDR *Providencia* species and eight XDR *Providencia* species and found sensitivity to aztreonam-avibactam at MIC 0.5 μg/ml. They concluded that aztreonam-avibactam may represent a valuable option for treating infections caused by CRE, MDR and XDR Enterobacteriaceae, including OXA-48 and MBL-producing strains [16]. Chew et al. observed that aztreonam-avibactam combination can also be used in dual-carbapenemase-producing Enterobacteriaceae to inhibit MβLs (NDM, IMP, or VIM MβLs) with coexistent KPC or OXA-48-like carbapenemases [17].

Hackel et al. tested 1521 isolates of Enterobacteriaceae intrinsically resistant to colistin and found 100% sensitive to ceftazidime-avibactam [18]. Jayol et al. tested 63 colistin resistant *Klebsiella pneumonia* isolates, among which 32 were OXA-48 producers and suggested ceftazidime-avibactam is an effective therapeutic option for treating infections caused by colistin resistant and KPC or OXA-48 *Klebsiella pneumoniae*. In addition to that they also recommend ceftazidime-avibactam and aztreonam combination against colistin resistant and NDM-producing *K. pneumoniae* [19]. Petrosillo et al. described the use of ceftazidime-avibactam and fosfomycin for colistin resistant *K. pneumoniae*. They also emphasized on soon-to-be commercially available plazomicin and cefiderocol, as novel antimicrobial options and advised to consider the future use of innovative therapeutic strategies in development, including bacteriophages therapy and monoclonal antibodies [20]. OXA 48 producer, which is a global threat has now been detected in Nepal as well. Ceftazidime- avibactam combination or aztreonam -avibactam combination is the drug of choice which is not available in Nepal. Thus, knowledge on the occurrence of the OXA-48 producers may also encourage the pharmaceutical companies and The Ministry of Health to facilitate provision of drug of choice like ceftazidime-avibactam in Nepal.

Since OXA-48 enzyme leads to carbapenem resistance, we are obliged to use colistin and tigecycline due to unavailability of drug of choice in our country. However, some organisms belonging to tribe Proteeae of family Enterobacteriaceae (Proteus, Providencia and Morganella) are intrinsically resistant to colistin and tigecycline. Therefore, organisms which are OXA-48 producers and intrinsically resistant to colistin when infect patients causes high mortality. Use of colistin for colistin susceptible organisms but OXA- producer can help treat the infection, although it may result in adverse effects like renal toxicity. OXA-48 carbapenamase producers are a rising threat to the whole world so that their routine detection is a must. The potential benefits identifying the resistance pattern like OXA producer include initiation of early appropriate therapy in carbapenemase producing isolates.

Through this paper we alarm the official and governmental agencies to focus on the drug resistance mechanisms and to make arrangements for the provision of drugs like ceftazidime+ avibactam or aztreonam + avibactam in Nepal because carbapenamase producing pathogens with intrinsic colistin resistance can be fatal in the resource limited countries where new avibactam-based antibiotic combinations are not available. Protocols should be generated to make this drug available just for the prescription of ID physicians. In addition, developing infectious diseases expert academic programs in the medical institutes of Nepal is needed.

In conclusion, carbapenamase producing bacteria such as MBL, KPC, AmpC and co-producers are frequently detected in our laboratory and in the country. Unfortunately, phenotypic detection of OXA-48 is not routinely practiced. Since, we have detected OXA-48, which results in resistance to almost all antibiotics, drug resistance patterns should be characterized in each case by standard phenotypic method or by Xpert Carba-R assay for appropriate antibiotic selection and better patient care. Also larger studies are required to know the exact burden of OXA 48 producer in Nepal.

## Data Availability

Data generated or analyzed during this study are included in this published article and remaining are available from the corresponding author on reasonable request.

## Abbreviations

AmpC: class C cephalosporinase
CLSI: Clinical and Laboratory Standards Institute
EDTA: Ethylene diamine tetraacetic acid
ESBL: Extended spectrum beta-lactamase
KPC: *Klebsiella pneumoniae* Carbapenamase
MBL: Metallo-beta-lactamase MHT Modified Hodge Test
NDM: New Delhi metallo-beta-lactamase
OXA-48: Oxacillinase-48
PBA: Phenyl Boronic Acid
